# Evaluation of Bronchiolitis in the Pediatric Population in the United States of America and Canada: A Ten-Year Review

**DOI:** 10.7759/cureus.43393

**Published:** 2023-08-12

**Authors:** Olamide O Ajayi, Afomachukwu Ajufo, Queen L Ekpa, Peace O Alabi, Funmilola Babalola, Zainab T. O Omar, Medara Ekanem, Chioma Ezuma-Ebong, Opeyemi S Ogunshola, Darlington E Akahara, Sapana Manandhar, Okelue E Okobi

**Affiliations:** 1 Internal Medicine, Obafemi Awolowo College of Health Sciences, Olabisi Onabanjo University, Sagamu, NGA; 2 Internal Medicine and Pediatrics, All Saints University, Roseau, DMA; 3 General Practice, Conestoga College, Kitchener, CAN; 4 Pediatrics, University of Abuja Teaching Hospital, Abuja, NGA; 5 Epidemiology and Public Health, Texas Department of State Health Services, San Antonio, USA; 6 Pediatrics, Dubai Medical College For Girls, Dubai, ARE; 7 General Medicine, Babcock University Teaching Hospital, Ogun, NGA; 8 Internal Medicine, Angelic Care Hospital, Abuja, NGA; 9 Pediatrics, Mass General Hospital, Massachusetts, USA; 10 Medicine, Windsor University School of Medicine, Cayon, KNA; 11 Pediatric Medicine, Jiamusi University, Covington, USA; 12 Family Medicine, Larkin Community Hospital Palm Springs Campus, Miami, USA; 13 Family Medicine, Medficient Health Systems, Laurel, USA; 14 Family Medicine, Lakeside Medical Center, Belle Glade, USA

**Keywords:** influenza, covid-19, seasonalitly, pandemic, oxygen, epinephrine, children, pediatric, rsv, bronchiolitis

## Abstract

Bronchiolitis is a well-known viral infection among the pediatric population, significantly impacting hospitalization rates. The COVID-19 pandemic profoundly affected respiratory viral infections, including bronchiolitis, as various mitigation measures were implemented. In this study, we analyzed bronchiolitis cases during the pandemic and post-pandemic period, aiming to identify changes in management guidelines and their incidence and management over the last 10 years. Moreover, we explored the relationship between bronchiolitis and COVID-19, a virus that gained rapid notoriety worldwide. By analyzing data from pediatric populations in Canada and the USA, we sought to understand the role of varying seasons in the peak periods of bronchiolitis infections.

The comprehensive review's results will provide valuable insights into bronchiolitis dynamics within the context of the COVID-19 pandemic. Our aim is to better comprehend the interplay between bronchiolitis, COVID-19, and seasonal variations, ultimately contributing to a deeper understanding of this respiratory viral infection and informing future management strategies. Furthermore, these findings can assist healthcare professionals in preparing for and responding to potential fluctuations in bronchiolitis cases in the post-pandemic era.

## Introduction and background

Bronchiolitis is a viral infection and a leading cause of hospitalization in the pediatric population. The common culprits include respiratory syncytial virus (RSV), influenza virus, rhinovirus (RV), human metapneumovirus (HMPV), enterovirus (EV), human coronavirus (HCoV), human parainfluenza virus (HPIV), human adenovirus (HAdV), and human bocavirus (HBoV) [1-21]. The most common causative organism is the RSV [1-32]. Bronchiolitis frequently presents in the first two years of life, affecting more than one-third of children in this age group. It is associated with one in 13 hospital visits with high rates of hospitalization and reinfection [2-22]. Bronchiolitis typically affects the lower respiratory tract and the smaller airways' epithelial cells, leading to the overproduction of mucus [4]. During the pandemic, respiratory viral infections declined due to the protocols implemented to curb the spread of the coronavirus disease (COVID-19) [5,6] caused by the SARS-CoV2 virus.

Pathophysiology of bronchiolitis

Once the body gets infected with these bronchiolitis viruses, such as RSV, it spreads from the upper respiratory tract to the medium and small bronchi and bronchioles, causing inflammation of the epithelial lining. Thus, there is mucus production and cell necrosis. All these ultimately obstruct the airway resulting in wheezing [7,25,33]. The severity of the symptom, therefore, follows the pathophysiology. The mucus production from the epithelial inflammation activates the cough reflex to expel the mucus. This process is underdeveloped in infants/toddlers, resulting in difficulty breathing, increased work of breathing, and the use of accessory muscles for respiration, which often results in hospitalization following the need for respiratory support to prevent the severe complication of respiratory failure [8].

Justification

In recent years, particularly post-pandemic, bronchiolitis's incidence, prevalence, and management have evolved, notably due to the significant role played by COVID-19 in its occurrence in children and other age groups affected. Before the COVID-19 pandemic, there were 2.7 million annual deaths globally due to lower respiratory tract infections, with RSV and influenza being the leading causes of lower respiratory tract infections in children. RSV mortality reported among US infants and children is variable [6]. In children younger than two years, RSV infection presents as bronchiolitis, causing apnea and reduced oral intake in younger infants [6].

According to Zheng et al., RSV epidemics exhibit distinct spatiotemporal patterns in the United States, with predictable seasonal timing and duration [9]. However, after implementing mitigation measures for the COVID-19 pandemic in March 2020, the prevalence of RSV has decreased in many areas of the United States. The severity and length of these efforts varied by state. The low positivity rate persisted throughout the typical RSV season in the fall and winter of 2020 to 2021. Many other countries reported similarly low frequencies of RSV detection in the 2020 RSV season [9]. As mitigation measures were gradually withdrawn, different patterns of RSV epidemics emerged in other regions in early 2021. Small spring and summer waves of RSV activity were reported in France, Spain, and many US states [9]. Large out-of-season surges of RSV infections were reported in Australia, South Africa, and several southern US states [9].

In Canada, there was an apparent disappearance of the Respiratory Syncytial and influenza viruses following the pandemic. Meanwhile, SARS-CoV-2 and rhinovirus infections remained prevalent [6,1]. As a means to reduce avoidable medical visits, several RSV programs were abridged or paused, including the seasonal administration of monoclonal antibodies for RSV [6]. During the pre- and intra-pandemic period from August 25, 2019, to May 2, 2020, about 4.6% of RSV tests were positive compared to about 0.07% of positive test results in the inta-pandemic period from August 2, 2020, to May 9, 2020.6 However, a possible post-pandemic resurgence is anticipated due to relaxation of physical distancing measures, as seen recently in the interseason revival of the infection, similar to the emergence in the US as mitigation measures were lifted [6]. This is in the wake of presumed lower levels of pediatric immunity, especially in ages two years and below, due to lower exposures during the pandemic [6,29].

This paper evaluates the evolution of bronchiolitis and its management in the last decade, as well as the impact of COVID-19 and other factors on its incidence and prevalence. The significance of this is evident in the variability of seasonal presentation of bronchiolitis in children, especially within the first two years of life; irrespective of the causative agent, there were apparent changes in the known seasonal patterns following the COVID-19 pandemic.

## Review

Analysis and comparison of seasonal bronchiolitis peaks before, during, and after the pandemic

Bronchiolitis is a significant public health concern worldwide and typically exhibits seasonal patterns. Bronchiolitis, mainly caused by RSV, follows well-documented seasonal peaks that tend to remain consistent yearly within a given country. Various factors contribute to the seasonality of bronchiolitis. One key element is the behavior of the RSV, which tends to thrive and spread more efficiently in colder temperatures [10,26]. The winter season provides favorable conditions for the survival and transmission of the virus, leading to increased cases of bronchiolitis [10,26].

Additionally, there are increased indoor gatherings and travels during colder months. Subsequently, increased proximity among individuals facilitated the spread of respiratory infections, including bronchiolitis, during the pre-COVID-19 era [6,10,11]. While the aforementioned seasonal patterns may have been observed in many regions, it's essential to recognize that local variations occur where some areas experience slightly different peak periods or exhibit unique regional factors that influence the seasonality of bronchiolitis [8,10,26]. This understanding of the seasonal patterns of bronchiolitis is beneficial to healthcare providers, public health officials, and parents as it helps in preparedness for increased healthcare demand during peak periods, allocation of resources, and implementation of preventive measures.

Before the COVID-19 pandemic, the seasonal peak periods of bronchiolitis (specifically RSV-related cases) in the United States and other regions with similar climates typically began in the fall. They reached their highest point during winter. The season's onset varied, with cases emerging between mid-September and mid-November. Following the outset, bronchiolitis cases steadily increased, eventually peaking between late December and mid-February [8,10]. Healthcare facilities experienced a surge in bronchiolitis-related hospitalizations and mortality during this peak [9,12,27]. As the winter season transitioned into spring, there was a gradual decline in bronchiolitis cases observed. These seasonal drops usually happened from mid-April to mid-May, marking the end of the peak period [9,12,27]. However, the exact timing and duration of the seasonal patterns notably varied across different regions of the United States. They may have been influenced by climate, viral circulation, and population density [9,27].

The emergence of the SARS-CoV-2 virus in December 2019 and the subsequent COVID-19 pandemic led to significant changes in the seasonal occurrence and peaks of bronchiolitis, including the cases caused by respiratory syncytial virus (RSV) and influenza [1]. In response to the global health crisis, various non-pharmaceutical interventions were implemented to slow the transmission of the virus. These interventions included increased hand hygiene, sanitizers, isolation and quarantines, travel restrictions, workplace and school closures, physical and social distancing, and mask-wearing [1]. While the primary goal of these interventions was to control the spread of SARS-CoV-2, they also had unintended consequences on the dynamics and transmission of other respiratory viruses [1]. These implemented non-pharmaceutical interventions remarkably impacted the transmission of respiratory viruses, particularly RSV and influenza, among infants and young children [1]. The measures effectively reduced the transmission of these viruses due to their similar modes of communication and susceptibility to the implemented interventions [1,10].

As a result of the COVID-19 mitigation measures, there was a noticeable decrease in RSV and influenza cases in many countries [8,10,13]. For example, in Canada, a study reported a significant decline in RSV cases during the pandemic compared to the previous year [8]. Pascal et al. further highlighted a study done and written by the Centre for Immunization and Respiratory Infectious Diseases which indicated that out of 339,627 tests for RSV, only 239 were positive between August 29, 2020, and May 8, 2021 [8]. In comparison, 412,861 tests for RSV were reported over a similar period in the previous year (August 25, 2019, and May 2, 2020), of which 18,860 were positive[8]. The incidence of RSV continued to decline rapidly following the introduction of COVID-19 mitigation measures in March 2020. This decline continued throughout the typical RSV peak seasons in the fall and winter of 2020 to 2021 and remained relatively low from May 2020 to March 2021, even as restrictions were gradually lifted [13,10].

In May 2020, the United States reported a remarkable decrease in influenza hospitalization rates compared to the low-severity influenza season of 2011/2012. The rates were estimated to be 90% lower. This trend persisted throughout the autumn and winter of 2020/2021.16 Similar observations were made globally, with the World Health Organization (WHO) and European Centre for Disease Prevention and Control (ECDC) reporting that influenza activity during the 2020/2021 season did not increase above baseline levels [10,9].

The relaxation of physical distancing measures and decreased circulation of SARS-CoV-2 in many developed countries led to unexpected surges in RSV cases in the early post-COVID period [8]. Accumulating susceptible individuals and reintroducing external infections may have triggered these out-of-season or interseasonal outbreaks, particularly in the spring and summer of 2021 [8]. The patterns varied among regions, with some experiencing small waves of RSV activity while others reported large surges [8].

In the United States, the Centers for Disease Control and Prevention (CDC) issued a health alert in June 2021 regarding increased interseasonal RSV activity in the southern states since March 2021, which is unusual for that time of year. This elevated interseasonal activity deviated from the typical circulation patterns of RSV. Similarly, Canada anticipated a similar resurgence of seasonal respiratory viruses during the summer of 2021 [10,34]. The WHO and ECDC reported that influenza activity during the 2020/2021 season did not increase above baseline levels, with no indication of an autumn/winter spike. This trend continued into the spring and summer of 2021 [8,14,15] (Table 1).

**Table 1:** Comparison of seasonal bronchiolitis peaks before, during, and after the pandemic This table is an original creation of the authors of this manuscript.

Period	Season onset	Season peak	Season offset
Pre-COVID	Mid-September to mid-November	Late December to Mid-February	Mid-April to mid-May
COVID	Disrupted	Varied	Disrupted
Post-COVID	Earlier than usual resurgence noticed around March-June 2021	November 2021	December 2021 to January 2022

Moreover, the bronchiolitis season changed its timing and duration during the post-COVID period. It started earlier than usual, reaching a peak in November 2021, followed by a gradual reduction between December 2021 and January 2022 [9,16,17]. This monthly distribution differed from the pre-pandemic seasons, where RSV, the most common cause of bronchiolitis, was active from November to the end of April, with a peak occurring from late December to mid-February [16,18].

Further studies conducted post-COVID revealed differences in the seasonal peaks of RSV, human rhinovirus-enterovirus (HRE), and parainfluenza viruses (PIV) compared to the pre-pandemic period. RSV, which previously peaked in winter and autumn, showed no clear peak during the post-COVID period [14,18]. The height of HRE, however, shifted to autumn and spring, whereas it was previously observed in winter. Influenza virus A had two peaks in summer and autumn during the post-COVID period, while the peak of the parainfluenza virus shifted to winter and summer [14,18].

Diagnosis and management of bronchiolitis

The diagnosis of bronchiolitis is mainly clinical and is based on a focused history and physical examination in patients with a high index of suspicion of the disease [1]. Bronchiolitis could present with symptoms similar to other diseases; the main goal in the history and physical examination is to differentiate infants with probable bronchiolitis from those with other respiratory illnesses and identify them [2]. Additionally, risk factors for progression to severe disease should be identified, and an assessment of disease severity should be made as this would guide the management [3].

Evidence-based studies and reviews have shown that further testing may result in unnecessary intervention and hospitalization [19,28]. The decision to hospitalize bronchiolitis patients depends on several considerations, including the social and medical aspects; a history of apneic episodes, signs and symptoms of severe respiratory distress, inability to tolerate oral hydration and maintain oxygen saturation, and progression of symptoms over the first 72 hours are the common suggestions for further monitoring and admission [10,11]. In infants with moderate to severe bronchiolitis, initial SpO2 was the best predictor of hospital admission and longer length of stay [20]. Efforts to better define and manage hypoxemia in bronchiolitis may be helpful. Following high-quality, evidence-based practice studies, bronchiolitis management is primarily based on general supportive measures, hydration maintenance, and oxygen supplementation [19,28]. The Canadian Pediatric Society's recommendation regarding using an epinephrine nebulizer alone or simultaneously with dexamethasone was equivocal. However, bronchodilator use was not recommended [3]. Palivizumab, an intramuscular injectable monoclonal antibody, can prevent bronchiolitis in infants with high-risk factors for RSV infection. Palivizumab is also used in ex-preterm babies and is proven to decrease RSV bronchiolitis admission and intensive care unit needs in this group [19,29].

History includes two to three days of viral prodrome of cough, fever, rhinorrhea [1], the first episode of wheezing before 12 months; however, wheezing is not always present [1], and a history of contact with an adult with a cough may or may not be present [1]. History of underlying risk factors such as underlying congenital heart disease, lung conditions, in-utero exposure to smoke, prematurity, and immune deficiency disorders are also important parameters in history [1,33].

Physical examination depends on the severity of the illness; children could present with varying clinical signs of respiratory distress [3]. Symptoms include tachypnea, intercostal and subcostal retractions, accessory muscle use, nasal flaring, grunting, cyanosis, apnea, wheezing or crackles, and low O2 saturations [1].

Overview of pediatric bronchiolitis guidelines in the United States

The American Academy of Pediatrics (AAP) provides the primary guidelines for pediatric bronchiolitis in the United States. In November 2014, the AAP published an updated clinical practice guideline, revising and reviewing the previous 2006 guideline. This guideline focuses on diagnosing, managing, and preventing bronchiolitis in children aged one to 23 months. The recommendations in the guideline focus on evidence-based reporting.

Diagnosis

Bronchiolitis should be diagnosed based on history and clinical presentation, characterized by lower respiratory symptoms such as cough, Tachypnea, wheezing, and increased respiratory effort, such as grunting, in children younger than two years. Additionally, risk factors for severe disease should be assessed, and an estimate of disease severity (increased respiratory rate, retractions, decreased oxygen saturation) should also be made. Further testing, including radiological or laboratory investigations, should not be routinely carried out unless expressly warranted [21,30].

Treatment

Supportive care: The mainstay of treatment should be supportive care, including adequate hydration and maintenance of oxygen saturation. Nasal suctioning is recommended to clear nasal secretions, especially in infants with difficulty feeding due to nasal congestion. Nebulized hypertonic saline may be administered to infants and children hospitalized for bronchiolitis [21,30].

Pharmacologic therapy: Bronchodilators (such as albuterol), epinephrine, and systemic corticosteroids should not be administered to treat bronchiolitis. Antibiotics should not be routinely administered but may be considered in cases of concurrent bacterial infections [21,30].

Oxygen therapy: Supplemental oxygen should be provided to maintain oxygen saturation above 90%. In severe cases, hospitalization may be required for closer monitoring and oxygen support [21,30].

Prevention

The guidelines emphasize the importance of preventive measures, such as hand hygiene with hand-washing or alcohol-based rubs, cessation of smoking by caregivers, and the need for exclusive breastfeeding for infants [21,30].

Palivizumab, a monoclonal antibody, may be considered for specific high-risk infants, i.e., infants with hemodynamically significant heart disease or chronic lung disease of prematurity, to prevent severe RSV infection. A maximum of five monthly doses should be given [21].

Overview of pediatric bronchiolitis guidelines in Canada

The Canadian Pediatric Society (CPS) has established the following recommendations for the diagnosis, treatment, and need for hospital admission.

Diagnosis

Bronchiolitis is a clinical diagnosis based on history and a physical examination [1]. Laboratory and radiologic investigations are not recommended in typical cases of bronchiolitis, as there has been no clinical evidence to show the benefits of such tests in aiding in diagnosis or improving treatment quality [1].

Management 

Management is mainly supportive, including hydration, minimal handling, gentle nasal suctioning, and oxygen therapy [1]. Oxygen saturation monitoring is recommended. Continuous monitoring is recommended in high-risk children, and intermittent monitoring is recommended for low-risk children [1]. Nasogastric (NG) tube feeding or IV fluid for hydration is recommended; recommended IV fluids include 0.9% NaCl or 5% D/S [1]. Epinephrine is not recommended in typical cases, and when used in emergency cases, patients should be monitored closely. Epinephrine use should be discontinued in cases of no clinical improvement. No clinical evidence supports using hypertonic saline, nebulized salbutamol, and steroids in routine instances. Antibiotics are not recommended unless there is a strong clinical suspicion of co-existing bacterial infection [1].

Decision to admit

The decision to admit considers the following factors; the infant's hydration status depends on the child's ability to maintain adequate hydration and hydration status at presentation. Respiratory quality is assessed using the respiratory rate and accessory muscles for respiration. Supplemental oxygen therapy is needed to maintain oxygen saturation >90%. Infants at risk for severe illness, which includes; Immunodeficiency, infants <3 Months, preterm delivery <35 weeks [1].

Comparison

Both sources emphasize the importance of clinical diagnosis based on history and physical examination findings. They also highlight the limited role of diagnostic testing in most cases. The CPS and AAP guidelines provide specific recommendations and management strategies.

Regarding strengths, the CPS guidelines provide comprehensive recommendations and consider the needs of vulnerable populations. The AAP guidelines stand out for their evidence-based approach, criteria for hospitalization, and prevention strategies [1,21] (Table 2).

**Table 2:** Comparison and evaluation of the guidelines for evaluating bronchiolitis in the pediatric population This table is an original creation of the authors of this manuscript.

Source	Strengths	Weaknesses
Canadian Pediatric Society (CPS)	Provides a detailed overview of the clinical presentation and natural history of bronchiolitis.	Does not extensively discuss specific diagnostic tests or their indications.
	Offers practical recommendations for the management of bronchiolitis in different healthcare settings.	
	Considers the needs of vulnerable populations and guides their management.	
American Academy of Pediatrics (AAP)	Presents evidence-based recommendations supported by a comprehensive review of the literature.	Some recommendations may not be applicable in resource-limited settings.
	Provides criteria for hospitalization and guidance on the use of pharmacologic therapies.	
	Includes preventive measures, such as palivizumab prophylaxis for high-risk infants.	

Recommendations

Bronchiolitis is diagnosed clinically based on a patient's medical history and physical exam. Usually, diagnostic procedures such as chest radiographs, blood tests, and viral or bacterial cultures are not advised. Clinical judgment should be used to determine whether to admit a patient to the hospital, considering their respiratory condition, their ability to keep enough hydration, and their family's ability to manage at home. The cornerstones of management include supportive care, fluids, gentle nasal suctioning, and oxygen therapy [22].

There is no difference between the NG and IV methods for supplementary hydration. An isotonic solution (0.9% NaCl/ 5% dextrose) and regular monitoring of serum Na are advised when IV fluids are utilized. It is not recommended to use epinephrine in typical circumstances. If epinephrine inhalation is tried as a trial in the emergency room, continued care should start once there are apparent signs of improvement. Salbutamol (Ventolin) is not advised in ordinary cases of bronchiolitis, and there is currently insufficient data to justify the use of hypertonic 3% sodium chloride in such circumstances. It is not advised to use corticosteroids in routine instances. Antibiotics are not recommended unless substantial evidence of an underlying bacterial illness exists. Chest physical therapy is not suggested [22].

It is advised to practice oxygen saturation monitoring carefully when treating hospitalized patients. While intermittent monitoring or periodic evaluations are appropriate for lower-risk children and patients showing clinical improvement, continuous saturation monitoring may be necessary for high-risk children throughout the acute stage of the illness [22].

## Conclusions

Bronchiolitis is a significant public health concern and largely contributes to the global burden of respiratory viral infections with known seasonality variabilities in North American nations. Evaluating bronchiolitis in children in the USA and Canada has reinforced this knowledge from available evidence and, notably, the effect of the COVID-19 pandemic on these seasonal variations. Bronchiolitis is a viral inflammation of the bronchioles most commonly caused by RSV in children and is also a leading cause of hospitalization in this population. Diagnosis is clinical, and management is mainly supportive of maintaining oxygen saturation. American and Canadian guidelines do not recommend routine investigations to diagnose the disease in typical presentations; however, laboratory and radiological investigations may be indicated in exceptional cases. The emergence of the SARS-CoV-2 pandemic (COVID-19) in 2019 tweaked the usual seasonal peaks of bronchiolitis viruses. Non-pharmaceutical interventions such as hand hygiene and social distancing, enforced during the pandemic, remarkably reduced the transmission of respiratory viruses notorious for bronchiolitis in infants and young children, particularly RSV and influenza. Relaxation of COVID-19 mitigation measures post-pandemic in June 2021 saw a simultaneous surge in interseason RSV cases.

Comparing the guidelines in the USA and Canada, we concluded that bronchiolitis remains a clinical diagnosis with a limited role for testing except in exceptional circumstances like those outlined, and management is mainly supportive. Finally, our study showed that despite the guidelines set in place to limit the spread of this disease, there are still areas needing improvement. There is no specific method to protect vulnerable pediatric populations from contracting the viruses, thus reinforcing the need for an RSV vaccine. Healthcare professionals must also continue public health advocacy of hand hygiene and vaccination against other known viral culprits to reduce further the spread of circulating interseasonal bronchiolitis viruses in the general population.
